# Refractory erythema annulare centrifugum treated with roflumilast

**DOI:** 10.1016/j.jdcr.2024.02.004

**Published:** 2024-03-21

**Authors:** Pamela Calderon, Hamza Ajmal, Mitchell Brady, Francisca Kartono

**Affiliations:** a Department of Dermatology, Corewell Health Farmington Hills, Farmington Hills, Michigan

**Keywords:** annulare, centrifugum, dermatology, EAC, erythema, roflumilast, zoryve

## Introduction

Erythema annulare centrifugum (EAC) is the most common of the major figurate erythemas, which also include erythema marginatum, erythema migrans, and erythema gyratum repens, being classified as a reactive erythema.^1^ It has been suggested that the epidermal spongiosis histologically represents a reaction to one of many antigens, of which could be infectious agents, drugs, Crohn’s disease, pregnancy, autoimmune endocrinopathies, and occasionally, neoplasms.^2^ Lesions present first as firm pink papules that expand centrifugally and develop central clearing, then enlarge centrifugally again to form a large plaque. Asymmetric plaques can appear polycyclic. The peak incidence for EAC is the fifth decade of life, however EAC can appear throughout all age groups, with no known gender predilection.^1^ EAC typically resolves with treatment of the underlying condition, however topical or systemic corticosteroids, antibiotics, or antihistamines have been used to treat the condition itself.

Roflumilast 0.3% cream is a topical phosphodiesterase-4 (PDE-4) inhibitor.^3^ It is currently Food and Drug Administration approved to be used as a treatment for plaque psoriasis, including intertriginous skin in patients ≥6 years of age.^3^ We report a case of EAC refractory to conventional treatments, successfully treated with roflumilast, which, to our knowledge, has not been previously reported in literature.

## Case report

**Fig 1:**
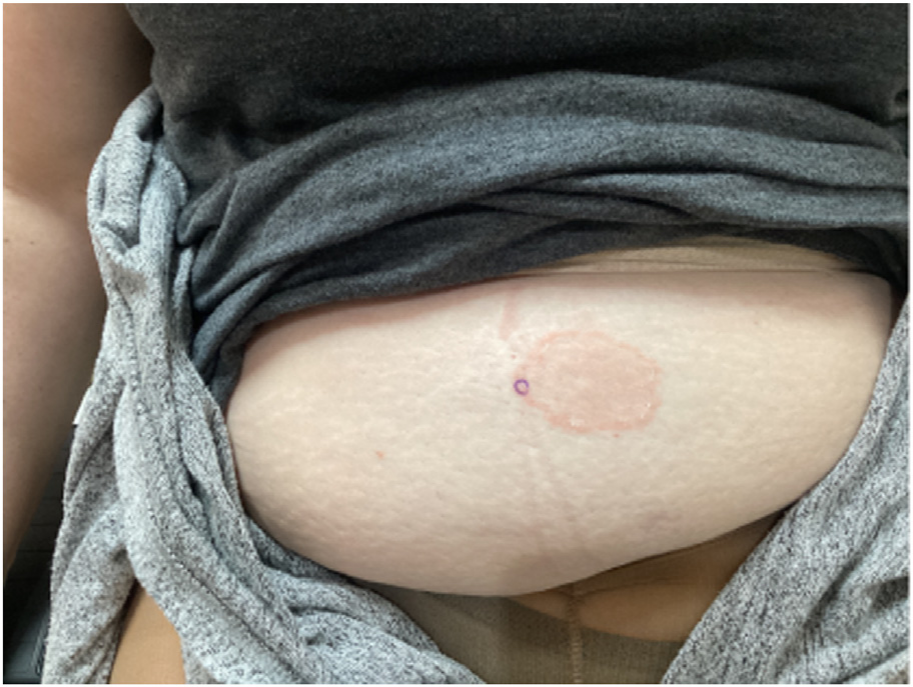
First patient encounter, an erythematous well demarcated 5 cm plaque with trailing scale.

A 46-year-old woman presented with a 2-month-old lesion on the stomach to our dermatology clinic. Her past medical history included cataplexy, narcolepsy, Hashimoto thyroiditis, high blood pressure, high cholesterol, polycystic ovarian syndrome, restless leg syndrome, herpes labialis, and diabetes mellitus. The patient had no record of starting any new medications or therapies, such as metronidazole, hyaluronic acid, or etanercept, which have been previously associated with EAC. She was referred by her primary care physician who suspected a fungal infection. Treatment with over-the-counter antifungal cream, ketoconazole cream, and triamcinolone ointment improved, but did not resolve the rash. At the time of our dermatology evaluation, the lesion was a solitary 5 cm erythematous plaque with a trailing scale on her mid abdomen (Fig 1).

A punch biopsy, potassium hydroxide examination, and skin scraping for fungal identification by polymerase chain reaction test were performed to determine a definitive diagnosis. Given the initial differential diagnoses of atopic dermatitis, tinea corporis, and EAC, samples of crisaborole, a topical PDE-4 inhibitor used to treat mild-to-moderate atopic dermatitis, were given to observe how the rash responded.

**Fig 2:**
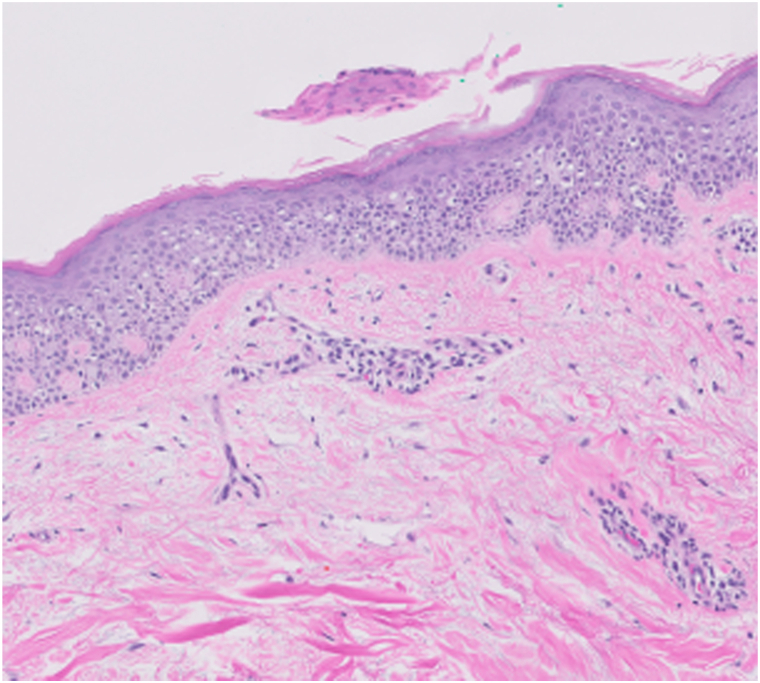
Slide ½ case CTM229169 ×10 confirming erythema annulare centrifugum (EAC) diagnosis, superficial perivascular Lymphocytic infiltrate, mild spongiosis, mound-like parakeratosis representing the superficial variant of EAC.

At a follow-up evaluation 2 weeks later, punch biopsy results revealing spongiotic dermatitis with a focal mound-like parakeratotic scale with findings compatible with EAC were discussed (Fig 2). Potassium hydroxide examination and polymerase chain reaction results were negative for fungal presence, including no detection of dermatophytic species, *Candida* spp, and *Malassezia* spp. With crisaborole, lesions persisted, and did not diminish in dimension. The patient was started on triamcinolone acetonide 0.1% cream twice daily for EAC treatment.

**Fig 3:**
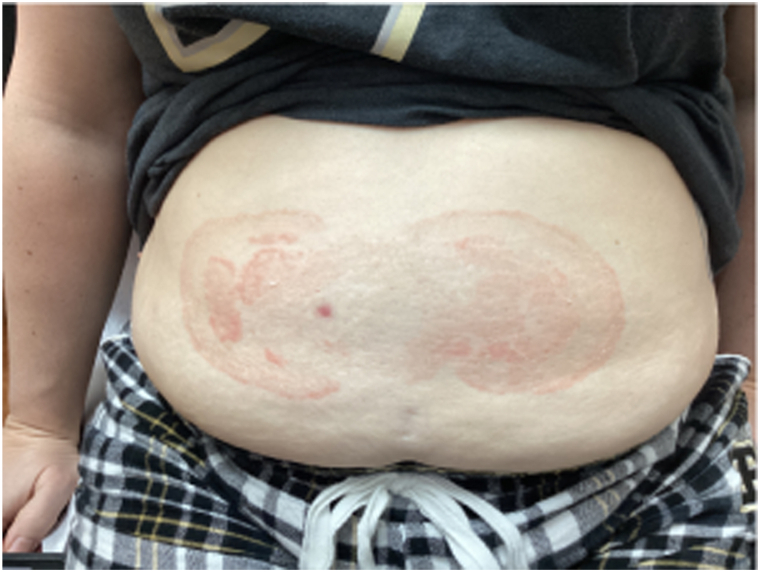
Third patient encounter, a large 30 cm polycyclic plaque with trailing scale and central clearing.

Four and half months later, despite consistently using triamcinolone acetonide 0.1% cream 3 to 4 times a day, the lesion had grown into an approximately 10 cm by 30 cm figurate erythematous polycyclic plaque with a trailing scale and central clearing (Fig 3). Patient was subsequently prescribed tacrolimus 0.1% ointment twice a day to use instead of triamcinolone.

As the lesions persisted, patient was advised to consult with her primary care physician for age appropriate cancer screening, as EAC has been associated with a paraneoplastic cause.^1^ Laboratory workup revealed supplementary negative abnormal blood work findings, ultrasound thyroid, and colonoscopy results.

**Fig 4:**
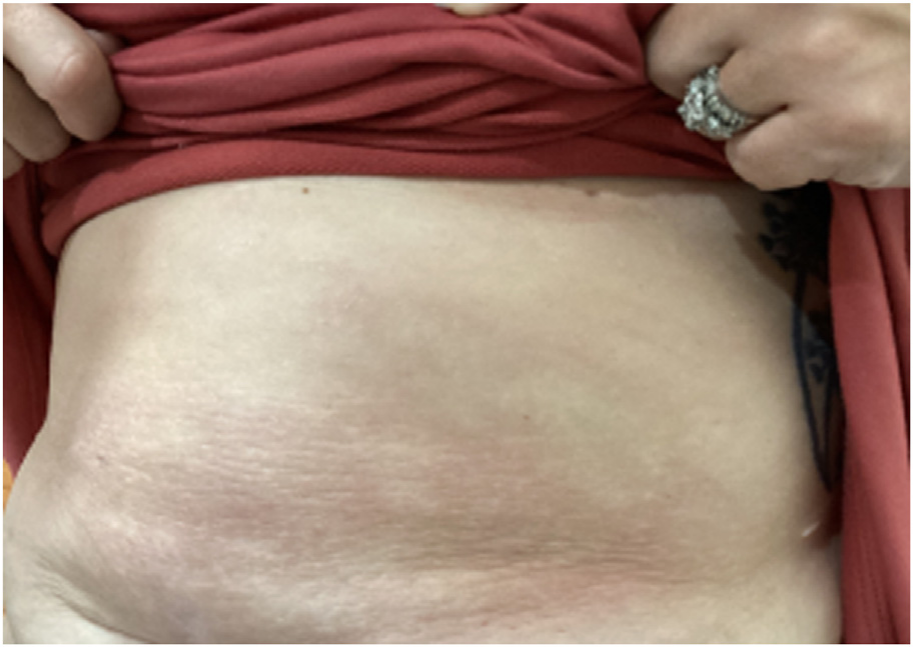
Erythema annulare centrifugum lesion cleared with postinflammatory hyperpigmentation.

At a 2-month follow-up visit, the patient’s rash remained refractory to topical tacrolimus. Topical roflumilast 0.3% cream twice per day was started. After only 1 month of usage, the lesion reached almost complete clearance. Two months later, all signs of EAC were gone (Fig 4), and clearance was maintained with usage of roflumilast 0.3% cream every other day. The patient did not suffer any adverse side effects from roflumilast application.

## Discussion

Presently, there is no consistent, singular treatment for EAC, because of the unpredictable and refractory nature of the disease. To date, it remains unclear what the pathophysiology is in the formation of EAC lesions.^4^ Patients with identifiable triggers, such as EAC secondary to disease or exposure, are treated conservatively by either treatment of the underlying disease or removal and or avoidance of the trigger, such as hypersensitivity reactions to external or internal stimulus, viruses, parasites, ectoparasites, bacteria, or various foods and drugs.^1^ Hashimoto thyroiditis was considered as a potential causative agent in our patient, although disregarded as her diagnosis was well controlled on medications and no correlations of any thyroiditis episode and EAC flares were found. Patients who do not have an identifiable trigger are treated with corticosteroids and antipruritic medications, such as topical calcipotriene and topical tacrolimus, for improvement of the skin lesion or narrow-band ultraviolet B, subcutaneous etanercept, subcutaneous interferon alfa, or oral metronidazole for symptomatic relief.^1^

Roflumilast is a selective PDE-4 inhibitor. As PDE-4 is a metabolizer of cyclic adenosine monophosphate, intracellular cyclic adenosine monophosphate is accumulated. In immune cells, high levels of cyclic adenosine monophosphate results in the suppression of the expression of proinflammatory cytokines, including tumor necrosis factor-alfa, interleukin 17, and interferon gamma.^5^ We hypothesize that EAC is most likely triggered or exacerbated by proinflammatory cytokines, which is diminished with the use of roflumilast.^6^ Roflumilast also has a significantly higher selectivity for PDE-4B, which is associated with antiinflammatory results, requiring a lower half-maximal inhibitory concentration to yield an effective dose, in comparison to crisaborole.^3^ Reduction in inflammation may have also been due to the induction of interleukin 10, which is an antiinflammatory cytokine.^7^ We believe that roflumilast provided clearance where crisaborole failed because *in vitro* studies have demonstrated roflumilast to be 25 to 300 times more potent than crisaborole based on half-maximal inhibitory concentration values. Half-maximal inhibitory concentration is a measure of drug potency in whole cell assays.^6^ The reduction of proinflammatory cytokines and the differentiation of antiinflammatory cytokines may have prompted the reduction of redness and EAC symptoms in this patient.

Roflumilast was successful in clearing EAC in this patient refractory to topical steroids, crisaborole, and calcineurin inhibitor usage, with no adverse side effects reported. Further controlled studies are needed to evaluate roflumilast as a novel and safe treatment option for EAC.

## Conflicts of interest

None disclosed.
